# Functional analysis of potential cleavage sites in the MERS-coronavirus spike protein

**DOI:** 10.1038/s41598-018-34859-w

**Published:** 2018-11-09

**Authors:** Hannah Kleine-Weber, Mahmoud Tarek Elzayat, Markus Hoffmann, Stefan Pöhlmann

**Affiliations:** 1 Infection Biology Unit, German Primate Center - Leibniz Institute for Primate Research, Kellnerweg 4, 37077 Göttingen, Germany; 2 Faculty of Biology and Psychology, Wilhelm-Weber-Str. 2, University Göttingen, 37073 Göttingen, Germany

## Abstract

The Middle East respiratory syndrome-related coronavirus (MERS-CoV) can cause severe disease and has pandemic potential. Therefore, development of antiviral strategies is an important task. The activation of the viral spike protein (S) by host cell proteases is essential for viral infectivity and the responsible enzymes are potential therapeutic targets. The cellular proteases furin, cathepsin L and TMPRSS2 can activate MERS-S and may cleave the S protein at two distinct sites, termed S1/S2 and S2′. Moreover, a potential cathepsin L cleavage site in MERS-S has been reported. However, the relative importance of these sites for MERS-S activation is incompletely understood. Here, we used mutagenic analysis and MERS-S-bearing vectors to study the contribution of specific cleavage sites to S protein-driven entry. We found that an intact S1/S2 site was only required for efficient entry into cells expressing endogenous TMPRSS2. In keeping with a previous study, pre-cleavage at the S1/S2 motif (RSVR) was important although not essential for subsequent MERS-S activation by TMPRSS2, and indirect evidence was obtained that this motif is processed by a protease depending on an intact RXXR motif, most likely furin. In contrast, the S2′ site (RSAR) was required for robust viral entry into all cell lines tested and the integrity of one of the two arginines was sufficient for efficient entry. These findings suggest that cleavage at S2′ is carried out by proteases recognizing a single arginine, most likely TMPRSS2 and cathepsin L. Finally, mutation of the proposed cathepsin L site did not impact viral entry and double mutation of S1/S2 and S2′ site was compatible with cathepsin L- but not TMPRSS2-dependent host cell entry, indicating that cathepsin L can process the S protein at auxiliary sites. Collectively, our results indicate a rigid sequence requirement for S protein activation by TMPRSS2 but not cathepsin L.

## Introduction

The family *Coronaviridae* comprises enveloped, positive sense RNA viruses that infect mammals (members of the subfamilies *Coronavirinae* and *Torovirinae*), birds (*Coronavirinae*) and fish (*Torovirinae*)^1^. Coronaviruses can have zoonotic potential and transmission of animal coronaviruses of the genus *Betacoronavirus* to humans has resulted in novel, severe respiratory diseases: The outbreak of severe acute respiratory syndrome (SARS; caused by SARS-related coronavirus, SARS-CoV) in Southern China in 2002 and its subsequent global spread were associated with almost 800 deaths, with the vast majority of cases occurring in Asia and Canada^2^. Although no new SARS cases were observed after 2004, another severe respiratory disease caused by a new betacoronavirus emerged in 2012: Middle East respiratory syndrome (MERS), caused by MERS-related coronavirus (MERS-CoV)^3,4^, was so far diagnosed in 2,229 patients and was responsible for 791 deaths^5^. The majority of cases were documented in the Middle East but the virus, like SARS-CoV, has been introduced into other countries via air travel and a MERS outbreak in South Korea was associated with more than 100 cases^6^. Importantly, MERS-CoV is still endemic in the Middle East and the virus may have pandemic potential. Therefore, it is important to devise novel antiviral strategies to combat MERS.

The MERS-CoV spike protein (MERS-S) is inserted into the viral envelope and mediates viral entry into target cells. For this, MERS-S binds to the cellular receptor dipeptidyl peptidase 4 (DPP4/CD26)^7^ via its surface unit, S1, and then employs its transmembrane unit, S2, to fuse the viral membrane with a host cell membrane, which allows the delivery of the viral genome into the host cell cytoplasm. However, receptor binding alone is not sufficient for S protein-driven entry. The S protein is synthesized as an inactive precursor and is converted into its active form upon cleavage by host cell proteases^8,9^. In fact, proteolytic processing of MERS-S might be sufficient to trigger the membrane fusion reaction and is subsequently referred to as activation. The host cell proteases responsible for MERS-S activation constitute potential targets for antiviral intervention and the identification of their cleavage sites might instruct the generation of inhibitors. Therefore, the proteolytic activation of MERS-S is in the focus of ongoing research endeavours.

The following host cell proteases can activate MERS-S in cell culture: Cathepsin L^10–12^, an endosomal, pH-dependent cysteine protease, furin^13,14^, a proprotein convertase expressed in the Golgi apparatus and to a lesser extent at the cell surface, and TMPRSS2^10,15^, a type II transmembrane serine protease that is believe to process the S protein and other substrates at or close to the cell surface. TMPRSS2 expression in target cells renders MERS-S-driven entry independent of the activity of cathepsin L^10,15^, indicating that during viral entry MERS-CoV makes contact with a TMPRSS2-positive compartment, most likely the plasma membrane, before it is trafficked into cathepsin L-positive endosomes. Cleavage of the S protein in the Golgi apparatus of infected cells has been proposed to be essential for subsequent MERS-S activation by TMPRSS2 or furin during entry into target cells because it may endow the S protein with sufficient structural flexibility to engage these proteases for processing^16^. Notably, activity of TMPRSS2 but not cathepsin L might be important for viral spread in the host. Thus, TMPRSS2 but not cathepsin L was found to be expressed at high levels in the respiratory epithelium^16,17^ and an inhibitor active against TMPRSS2 reduced SARS-CoV spread and pathogenesis in a rodent model while a cathepsin L inhibitor had little effect^18^. Moreover, activation by cathepsin L has been suggested to be a cell culture adaptation, at least in the context of the human coronaviruses 229E, HKU1 and OC43^19,20^. Finally, the contribution of furin to MERS-S activation in the host remains to be determined. In this context, it is noteworthy that one study questioned whether cleavage by furin contributes to MERS-CoV infectivity in cell culture^21^ while another showed that furin is not involved in S protein activation during host cell entry^22^.

**Figure 1:**
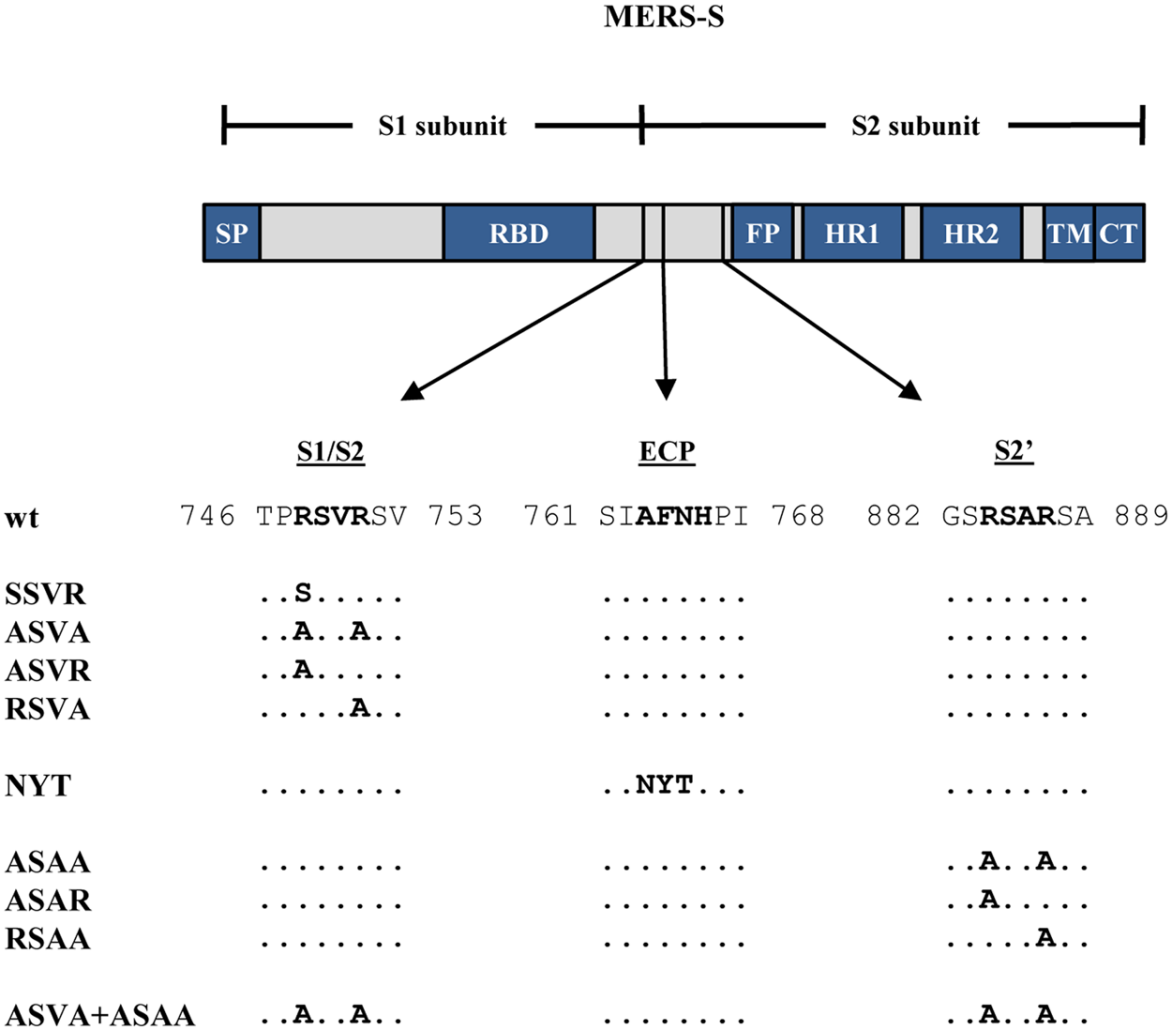
Domain organization and protease cleavage sites of MERS-S. MERS-S possesses two subunits, a surface subunit (S1) and a membrane-anchored subunit (S2). The S1 subunit harbors an N-terminal signal peptide (SP) and the receptor binding domain (RBD) while the S2 subunit contains domains required for membrane fusion, the fusion peptide (FP), two heptad repeats (HR1, HR2), and a transmembrane domain (TM). Moreover, the S2 subunit contains a cytoplasmic tail (CT). The S1/S2 cleavage site is located at the border between the S1 and S2 subunits while the S2′ site is located at the N-terminus of the FP. A proposed cleavage site for cathepsin L is located between the S1/S2 and S2′ sites (ECP, endosomal cysteine protease). The amino acid residues of the S1/S2, cathepsin L and S2′ sites are printed in bold and the mutations introduced into the cleavage sites are indicated.

The activation of MERS-S by host cell proteases requires S protein processing at defined sites. MERS-S harbors two cleavage sites found in all CoV S proteins: An S1/S2 site, composed of the amino acids RSVR, is located at the border between the S1 and S2 subunits, while an S2′ site, composed of the amino acids RSAR, is located upstream of the putative fusion peptide present within the S2 subunit (Fig. 1). The S1/S2 site is believed to be processed by furin in infected cells^14,21^ while the S2′ site should be processed during viral entry by all S protein activating proteases identified today, since generation of a free N-terminus of the fusion peptide is required for membrane fusion. Finally, an AFNH motif located between the S1/S2 site and the S2′ site was reported to be required for S protein-driven host cell entry and is believed to be processed by cathepsin L^23^. However, direct evidence that MERS-S activation depends on S protein processing at the S1/S2, cathepsin L and/or S2′ sites is frequently lacking. For instance, the sites required for S protein activation by TMPRSS2 are unknown. Moreover, it is largely unclear whether certain S protein activating proteases can use auxiliary sites in case S1/S2 and/or S2′ are not available.

Here, we performed mutagenic analysis to define the contribution of specific cleavage sites to MERS-S-driven entry into cell lines which expressed defined S protein activating proteases. Moreover, we used inhibitors to investigate protease choice. We found that the requirement for an intact S1/S2 site for MERS-S-mediated entry was cell type dependent while the integrity of the S2′ site was universally required. In contrast, mutation of the proposed cathepsin L site did not impact S protein-driven entry and we obtained evidence that this protease can use an auxiliary site for S protein activation if S2′ is not available. Finally, our results are compatible with the concept that the S1/S2 site is processed by proprotein convertases while the S2′ site is cleaved by TMPRSS2.

## Results

### The S2′ site but not the S1/S2 site is universally required for MERS S-driven host cell entry

**Figure 2:**
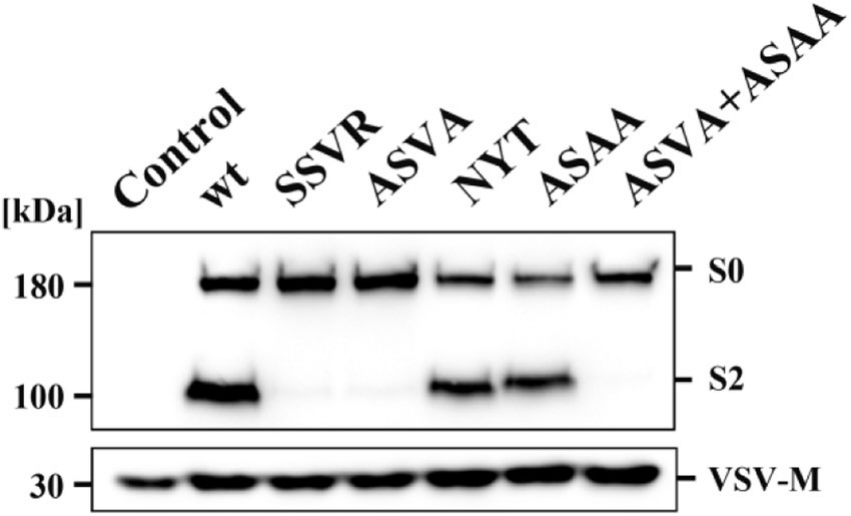
Incorporation of MERS-S proteins into rhabdoviral particles. Equal volumes of culture supernatants containing pseudoparticles harboring MERS-S wt or the indicated S protein mutants equipped with a C-terminal V5-tag were centrifuged and the pellets subjected to Western blot analysis, using an anti-V5 antibody. Arrow heads indicate bands corresponding to uncleaved precursor MERS-S (S0) and S2 subunit generated by cleavage at the S1/S2 border. The detection of VSV-M served as loading control. Shown is a representative Western blot from a total of twelve independent experiments

We first investigated whether the S1/S2, the cathepsin L and the S2′ cleavage sites (Fig. 1) were universally required for MERS-S-driven entry or were only required for entry into certain target cells. For this, we used a set of previously established S protein mutants^21,23^ and a VSV-based pseudotyping system. Mutants SSVR and ASVA harbored an altered S1/S2 site, the alteration in mutant NYT was previously shown to abrogate S protein activation by cathepsin L^23^ while mutant ASAA contained a mutated S2′ site (Fig. 1). Moreover, in mutant ASVA + ASAA both the S1/S2 and the S2′ site were altered (Fig. 1). We next employed a previously described VSV-based pseudotyping system^24^ to investigate how the mutations introduced into MERS-S modulated viral entry. For this, we first analyzed S protein incorporation into VSV particles produced in 293T cells. Immunoblot analysis revealed that all S protein mutants studied were efficiently incorporated into VSV particles, although some variation of incorporation efficiency was observed between experiments, and that mutation of the S1/S2 site largely abrogated proteolytic processing of the S protein in particle producing cells (Fig. 2), as expected^14,16,21^.

**Figure 3:**
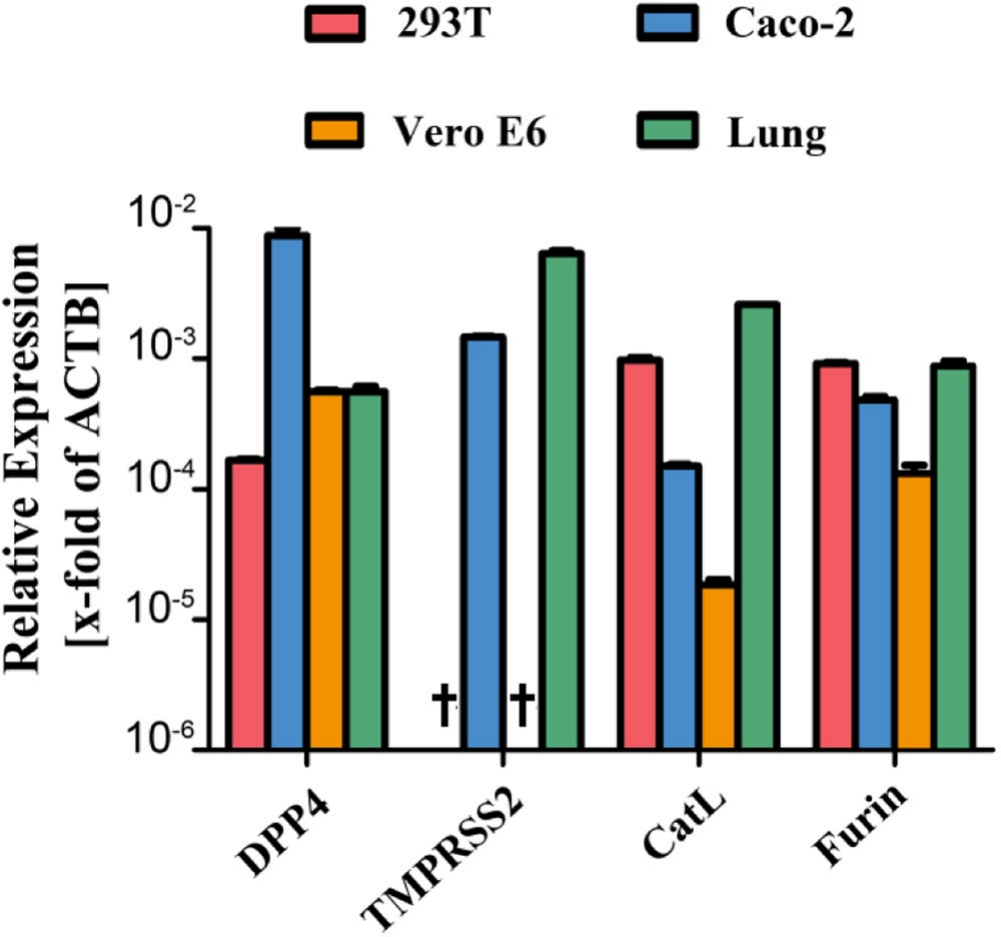
Expression of DPP4 and host cell proteases in target cell lines and lung tissue. Total cellular RNA was extracted from 293T, Caco-2 and Vero E6 cells, reverse-transcribed into cDNA and quantified for DPP4 and protease transcript numbers by quantitative RT-PCR. cDNA from human lung tissue was also included in the analysis. The numbers of DPP4/protease mRNA copies are shown relative to the housekeeping gene ß-actin (ACTB). Error bars indicate standard deviation (SD). Crosses indicate samples for which no transcripts were detected.

Next, we selected target cells for the analysis of MERS-S-driven entry. The human colon-derived cell line Caco-2, the human adrenal gland-derived cell line 293T (either untransfected or transfected to express high levels of DPP4) as well as the African green monkey kidney-derived cell line Vero E6 were chosen for analysis, since these cells were previously reported to be susceptible to MERS-S-driven entry and both Caco-2 and Vero E6 cells are known to allow amplification of authentic MERS-CoV^14,15,21,23^. Quantitative RT-PCR analysis of Caco-2, 293T and Vero E6 cells as well as human lung tissue, the natural target of MERS-CoV infection, revealed that Vero E6, 293T and lung tissue expressed roughly comparable amounts of DPP4 mRNA, while expression in Caco-2 cells was elevated (Fig. 3). In contrast, appreciable levels of TMPRSS2 mRNA were only detected in Caco-2 cells and lung tissue (Fig. 3), in keeping with published data^10,17^. Finally, mRNAs for cathepsin L as well as furin were readily detected in all cell lines tested and in lung tissue (Fig. 3). Thus, the cell lines under study were all positive for the MERS-CoV receptor, DPP4, but expressed different proteases known to be used by MERS-S for activation while expression of all proteases was robust in lung tissue.

**Figure 4:**
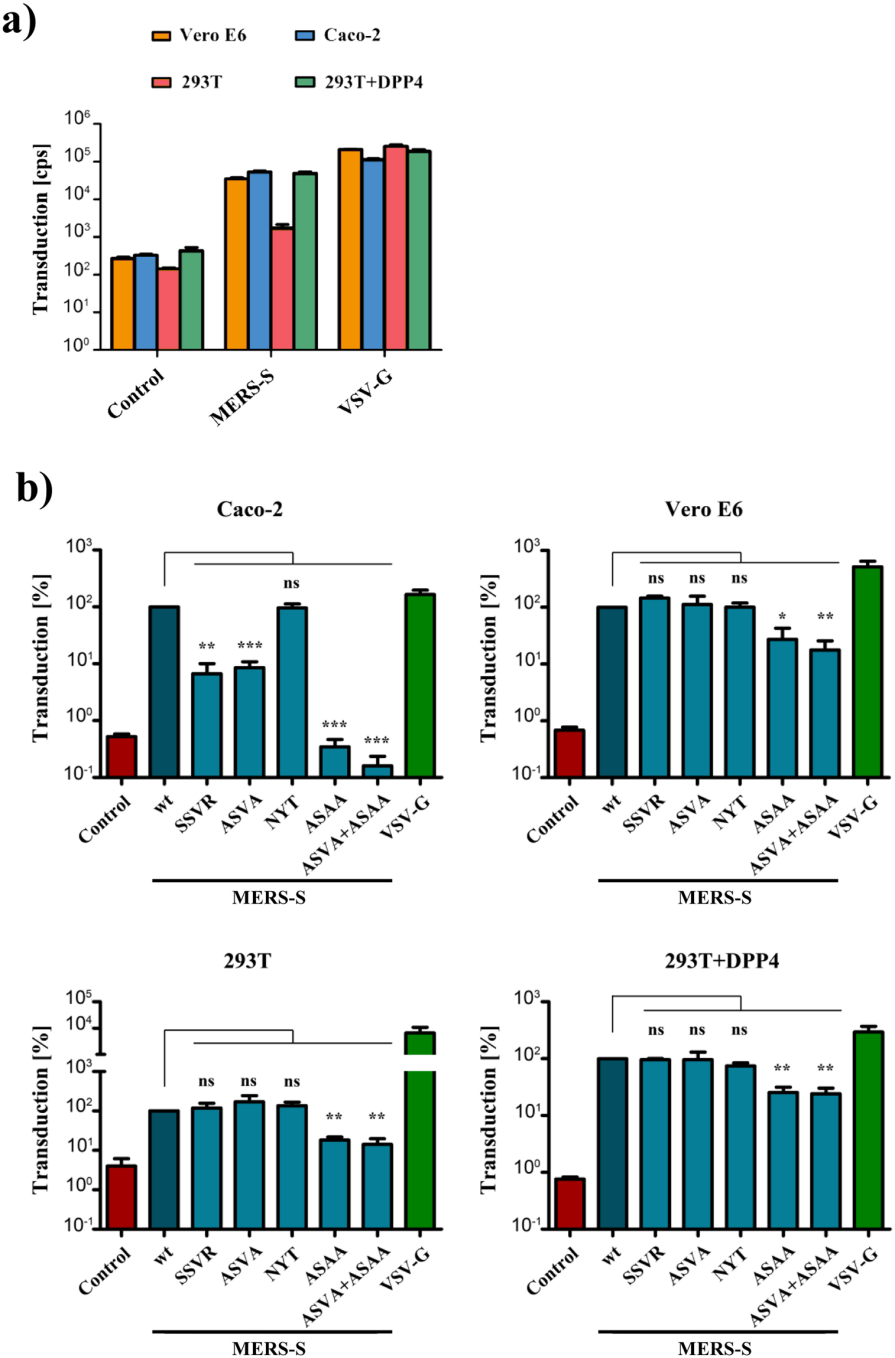
Requirement of the S1/S2 site for MERS-S-driven entry is cell type dependent, while the S2′ site is universally required. (**a**) Equal volumes of rhabdoviral vectors harboring MERS-S wt, the indicated S protein mutants, VSV-G or no glycoprotein at all (negative control) were used for transduction of Caco-2, Vero E6 cells or 293T cells that were either untreated or transfected with expression plasmid for DPP4. Transduction efficiency was quantified at 18 h post inoculation by measuring the activity of virus-encoded luciferase in cell lysates. Shown are the data from one representative experiment performed with quadruplicate samples, error bars indicate standard deviation (SD). Similar results were obtained in two separate experiments. (**b**) The combined results of three independent experiments carried out as described for panel a are shown. For normalization, transduction mediated by VSV particles harboring MERS-S wt was set as 100%. Error bars indicate standard error of the mean (SEM). Statistical significance of differences between transduction mediated by wt and mutant S proteins was analyzed using a paired, two-tailed students t-test (*p ≤ 0.05; **p ≤ 0.01; ***p ≤ 0.001; ns, not significant).

All cell lines under study were readily susceptible to transduction by control particles bearing VSV-G and were largely refractory to transduction by particles bearing no viral glycoprotein (Fig. 4a,b), as expected. Transduction mediated by wildtype MERS-S was comparable for Vero E6 and Caco-2 cells (~3 × 10^4^ c.p.s.) and generally higher as compared to untransfected 293T cells (~10^3^ c.p.s.) (Fig. 4a). However, when 293T cells were previously transfected with expression plasmid for DPP4, transduction levels were as high as for Vero E6 and Caco-2 cells (Fig. 4a). Transduction of target cells with particles bearing MERS-S revealed that alteration of the S1/S2 site markedly reduced entry into Caco-2 (approx. 12–15-fold reduction) but not 293T, 293T+DPP4 or Vero E6 cells (Fig. 4b). In contrast, an intact S2′ site was universally required for S protein driven entry, although the effects observed upon transduction of Vero E6, 293T and 293T+DPP4 were less pronounced than those seen for Caco-2 cells (Fig. 4b, roughly 300-fold reduction in Caco-2 as compared to 4–5-fold reduction in the other cell lines tested). Finally, mutation of the cathepsin L site had, contrary to published data^23^, no impact on S protein-driven entry into any of the cell lines studied.

### The S1/S2 and S2′ site in MERS-S are processed by different proteases

**Figure 5:**
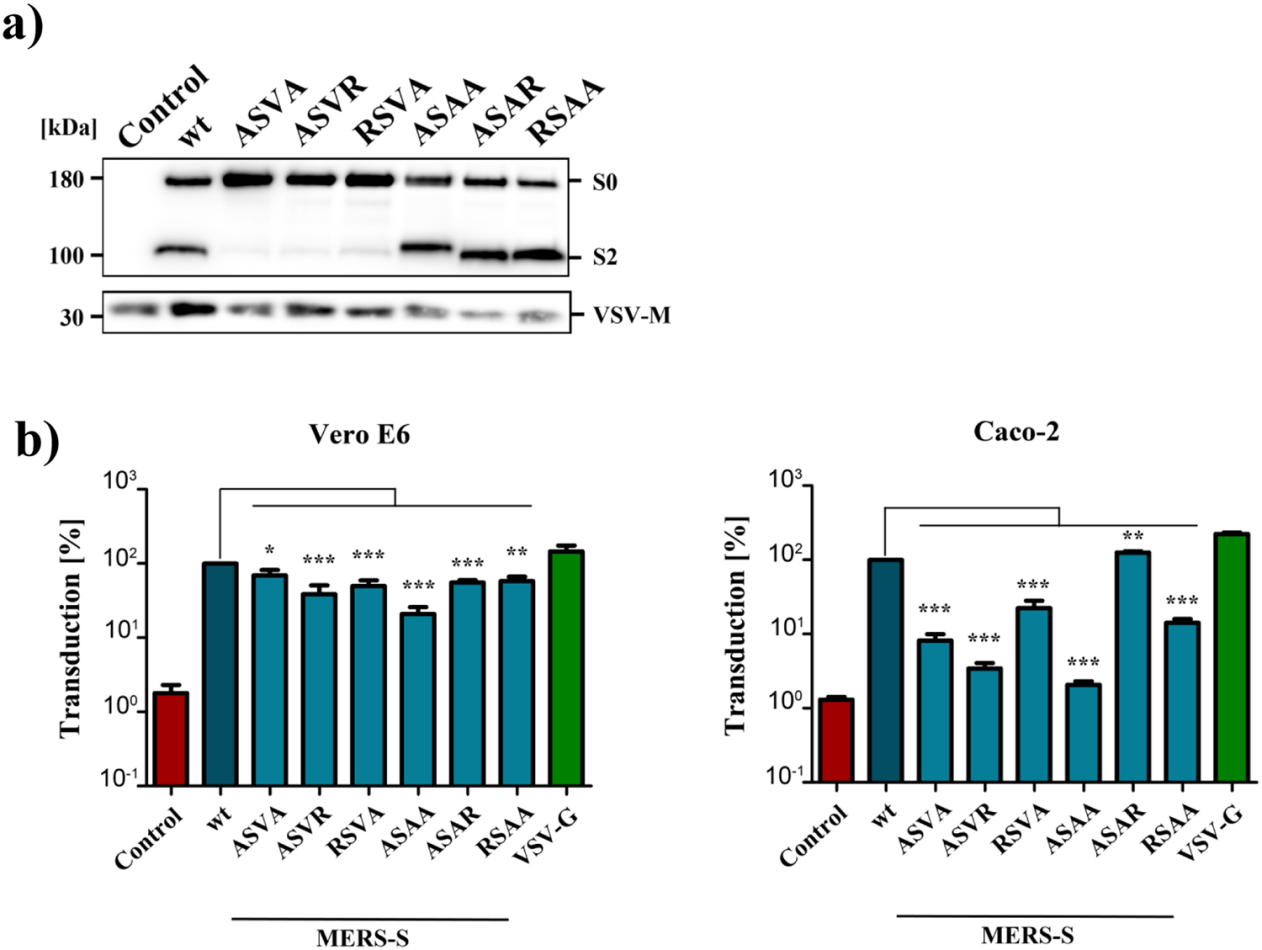
A single arginine at the S2′ site is sufficient for MERS-S activation. (**a**) Equal volumes of culture supernatants containing pseudoparticles harboring MERS-S wt or the indicated S protein mutants equipped with a C-terminal V5-tag were centrifuged and the pellets subjected to Western blot analysis, using an anti-V5 antibody. The results were confirmed in four separate experiments. (**b**) Vero E6 and Caco-2 cells were transduced with pseudoparticles harboring MERS-S wt, the indicated S protein mutants, VSV-G or no glycoprotein at all (negative control), and transduction efficiency was analyzed as described in the legend to Fig. 4. The average of nine separate experiments is shown, in which transduction mediated by MERS-S wt was set as 100%. Error bars indicate SEM. Statistical significance of differences between transduction mediated by wt and mutant S proteins was analyzed using a paired, two-tailed students t-test (*p ≤ 0.05; **p ≤ 0.01; ***p ≤ 0.001; ns, not significant).

We next determined whether the S1/S2 and the S2′ sites are cleaved by proteases exhibiting the same cleavage specificity or by enzymes that recognize different cleavage sites. Thus, both sites contain an RXXR motif, a prerequisite for cleavage by proprotein convertases, and could thus be processed by furin or related enzymes. Alternatively, these sites might be cleaved by proteases for which motifs encompassing a single arginine residue can be sufficient for cleavage, potentially cathepsin L and TMPRSS2. In order to discern between these two possibilities, we investigated entry driven by S protein mutants in which either one or both arginines were mutated. For this, we employed Vero E6 and Caco-2 cells as targets. Immunoblot revealed that all MERS-S mutants were efficiently incorporated into VSV particles (Fig. 5a) and none of the mutations introduced into the S1/S2 site markedly reduced entry into Vero E6 cells (Fig. 5b), in keeping with our findings stated above, although the 1.5–2.6-fold reduction observed in this set of experiments was statistically significant (note that the average of three experiments is shown in Fig. 4 while the average of nine experiments is shown in Fig. 5b). In contrast, mutation of a single arginine within this site was sufficient to reduce entry into Caco-2 cells by 4.5 to 29.4-fold (Fig. 5b), suggesting that the S1/S2 site is processed by a protease that depends on the presence of two arginines, like furin. Furthermore, the second but not the first arginine within the S2′ site was sufficient for efficient S protein-driven entry into Caco-2 cells while mutation of any single arginine at the S2′ site had only a minor, although statistically significant, effect on S protein-mediated transduction of Vero E6 cells (Fig. 5b). These results suggest that in Caco-2 and Vero E6 cells the S2′ site is mainly processed by a protease that requires a single arginine at its cleavage site, likely TMPRSS2 (Caco-2, see below) and cathepsin L (Vero E6).

### An intact S2′ but not S1/S2 site is essential for MERS-S activation by TMPRSS2

**Figure 6:**
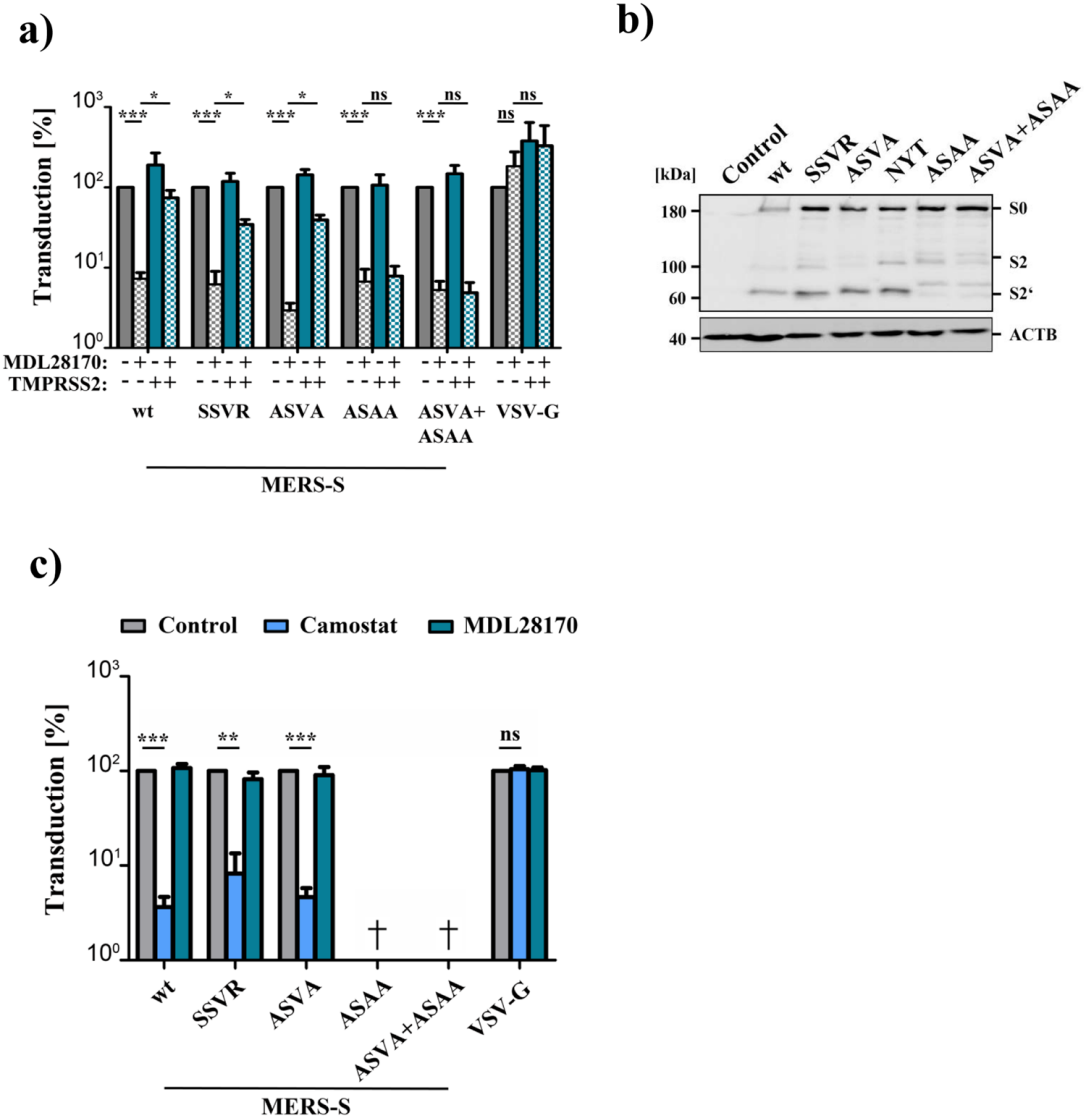
An intact S1/S2 site promotes but is not essential for MERS-S activation by TMPRSS2 (**a**) 293T cells were transfected with DPP4 plasmid or cotransfected with DPP4 and TMPRSS2 plasmid. At 24 h post transfection, cells were incubated with DMSO or cathepsin L inhibitor MDL28170 before being inoculated with pseudoparticles harboring MERS-S wt, the indicated S protein mutants or VSV-G. Transduction efficiency was quantified by measuring the activity of virus-encoded luciferase in cell lysates at 18 h post transduction. The average of three individual experiments is shown. Transduction of untreated, DPP4 transfected cells was set as 100%. Error bars indicate SEM. (**b**) 293T cells were cotransfected with TMPRSS2 plasmid and plasmids encoding MERS-S wt or the indicated S protein mutants equipped with a C-terminal V5-tag. Transfection of empty plasmid served as negative control. At 48 h post transfection, S protein expression in cell lysates was analyzed by Western blot. Bands representing uncleaved MERS-S (S0), the S2 subunit generated by cleavage at the S1/S2 site and an S2 fragment generated upon cleavage at the S2′ site are highlighted. Detection of ß-actin served as loading control. Similar results were obtained in two separate experiments. (**c**) Caco-2 cells were pre-incubated with the serine protease inhibitor camostat (bright blue) or the cathepsin L inhibitor MDL28170 (dark blue), or were control-treated with DMSO (gray). Subsequently, the cells were inoculated with equal volumes of preparations of pseudoparticles harboring MERS-S wt, the indicated S protein mutants or VSV-G. At 18 h post inoculation, transduction efficiency was quantified by measuring the activity of virus-encoded luciferase in cell lysates. The average of three independent experiments is shown. Transduction of control-treated cells was set as 100%. Crosses indicate samples for which no transduction above background levels was detected. Error bars indicate SEM. Statistical significance of differences between transduction of control-treated and inhibitor-treated cells was analyzed using a paired, two-tailed students t-test (**p ≤ 0.01; ***p ≤ 0.001; ns, not significant).

Our analyses had so far suggested that cleavage at S1/S2 is required for robust MERS-S-driven entry into Caco-2 cells, which express TMPRSS2. Therefore, we next asked whether cleavage at this site is also required for S protein-driven entry into cells in which only the TMPRSS2-activation pathway is operative. For this, we blocked MERS-S-driven entry into 293T+DPP4 cells by the cathepsin L inhibitor MDL28170 and asked whether this blockade can be overcome by expression of TMPRSS2. Entry driven by MERS-S wt and all S protein variants with mutations in the S1/S2 site was inhibited at least 13.7-fold by MDL28170 treatment and entry driven by MERS-S wt was fully rescued by directed expression of TMPRSS2 in target cells (Fig. 6a). Entry mediated by the S protein mutants with inactivated S1/S2 site was also rescued by TMPRSS2, although with reduced efficiency as compared to MERS-S wt (Fig. 6a), indicating that precleavage at S1/S2 is not an absolute prerequisite for subsequent S protein activation by TMPRSS2. In contrast, mutation of the S2′ site abrogated MERS-S activation by TMPRSS2. Moreover, analysis of MERS-S cleavage in a cis setting (i.e. protease and S protein expressed in the same cell) showed that mutation of the S2′ site resulted in aberrant cleavage products as compared to MERS-S wt and S protein mutants with alterations in the S1/S2 site (Fig. 6b). Finally, we asked whether the residual entry into the TMPRSS2^+^ Caco-2 cells observed upon mutation of the S1/S2 site was dependent on cathepsin L activity, as suggested by a previous study^16^, or was promoted by TMPRSS2. Preincubation of Caco-2 cells with protease inhibitors showed that entry driven by S proteins lacking an intact S1/S2 site was dependent on TMPRSS2 but not cathepsin L activity (Fig. 6c), indicating a dominant role of TMPRSS2 in S protein-driven host cell entry into Caco-2 cells. In sum, our data suggests that an intact S1/S2 site promotes but is not essential for MERS-S-activation by TMPRSS2.

### Residual Vero E6 cell entry observed upon mutation of the S1/S2 and S2′ site is promoted by cathepsin L activity

**Figure 7:**
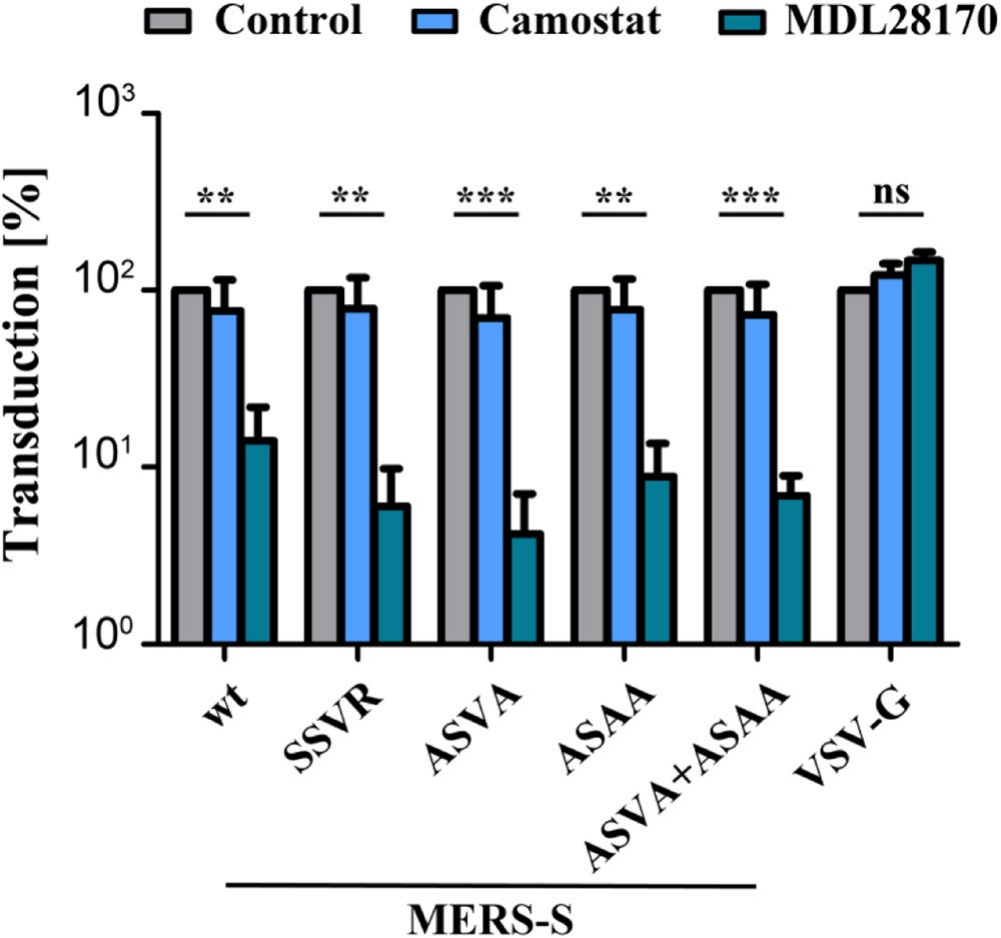
Cathepsin L activity is required for Vero E6 cell entry driven by a mutant S protein that lacks the S1/S2 and the S2′ sites. Vero E6 cells pre-incubated with serine protease inhibitor (camostat, bright blue), cathepsin L inhibitor (MDL28170, dark blue) or DMSO (control, gray) were inoculated with pseudoparticles as described for the Caco-2 cells in panel c of Fig. 6. At 18 h post inoculation, transduction efficiency was quantified by measuring the activity of virus-encoded luciferase in cell lysates. The average of three independent experiments is shown. Transduction of control-treated cells was set as 100%. Error bars indicate SEM. Statistical significance of differences between transduction of control-treated and inhibitor-treated cells was analyzed using a paired, two-tailed students t-test (**p ≤ 0.01; ***p ≤ 0.001; ns, not significant).

We found that simultaneous inactivation of the S1/S2 and S2′ site markedly reduced entry into Caco-2 cells, which depends on TMPRSS2, but only modestly reduced S protein-driven into Vero E6 cells, which depends on cathepsin L activity (Fig. 4). The latter finding raised the question whether the residual entry into Vero E6 cells was still dependent on cathepsin L activity. We addressed this question by analyzing transduction of Vero E6 cells which were preincubated with the cathepsin L inhibitor MDL28170 or the TMPRSS2 inhibitor camostat. Camostat did not reduce S protein-driven entry, as expected. In contrast, cell entry driven by all S proteins analyzed was comparably reduced upon MDL28170 treatment (Fig. 7). Remarkably, the cathepsin L dependence was also observed for mutants in which the S2′ was inactivated, indicating that cathepsin L, unlike TMPRSS2, can employ auxiliary cleavage sites for S protein activation.

## Discussion

MERS-CoV infection is associated with a case-fatality rate of 36% and the virus has pandemic potential^5^. Therefore, the identification of therapeutic targets is important and the cellular proteases responsible for S protein activation are candidates. However, cell line specific differences in protease choice and the sites in MERS-S that are processed by these proteases have not been fully elucidated. Our data support the concept that pre-cleavage of MERS-S at the S1/S2 site by furin promotes subsequent S protein activation by TMPRSS2^16^. However, pre-cleavage was not essential for S protein activation by TMPRSS2 and Caco-2 cell entry driven by a MERS-S variant lacking pre-cleavage was still TMPRSS2 dependent. Moreover, our results suggest that the S2′ site, specifically the second arginine, is important for efficient S protein activation by TMPRSS2 and indicate that cathepsin L but not TMPRSS2 can employ alternative sites for S protein activation. Collective, our results advance our understanding of the proteases facilitating S protein activation and of their cleavage sites within the S protein.

We employed a rhabdoviral pseudotyping system to study MERS-S-driven entry into target cells. This system and related ones have previously been shown to adequately mirror important aspects of CoV entry into target cells and allow studying this process without the need to work with infectious virus^25^. The wt S protein and all S protein mutants analyzed were readily incorporated into VSV particles, although some variations between particle preparations were observed. Uncleaved as well as cleaved forms of the S protein were detected in particles, with the exception of mutants with altered S1/S2 site, which exclusively incorporated uncleaved S protein. Thus, alteration of the S1/S2, cathepsin L and S2′ sites is compatible with particle incorporation of the S protein and only mutation of S1/S2 site abrogates S protein processing in particle producing cells. For the analysis of MERS-S-driven entry, well characterized cell lines were employed and two of them, Vero E6 and Caco-2, cells are permissive to MERS-CoV infection^21,26^. All cell lines analyzed as well as lung tissue expressed cathepsin L and furin mRNA, in keeping with the previously documented broad expression of these enzymes^27,28^, although cathepsin L levels in Vero E6 cells were relatively low. In contrast, robust levels of TMPRSS2 mRNA were only found in Caco-2 cells and lung tissue, again in agreement with published data^29^. Finally, all cell lines and lung tissue were positive for DPP4 mRNA, with Caco-2 cells expressing the highest levels, and transfection of DPP4 expression plasmid revealed that the low amounts of endogenous DPP4 in 293T cells limit the efficiency of S protein driven entry. In sum, well characterized particles as well as cell lines were employed to study the contribution of potential cleavage sites in MERS-S to proteolytic activation of the S protein.

The analysis of S protein mutants demonstrated that alteration of the S1/S2 motif markedly inhibited entry into Caco-2 cells but not the other cell lines studied. The requirement of S1/S2 for robust Caco-2 cell entry was perhaps not unexpected since only these cells expressed TMPRSS2 and a previous study reported that furin-mediated cleavage at S1/S2 in virus producing cells is required for subsequent S protein activation by TMPRSS2 during entry into target cells^16^. Our findings generally support this concept but suggest that certain aspects need to be reconsidered. Thus, in our hands pre-cleavage at S1/S2 promoted but was not essential for subsequent S protein activation by TMPRSS2, although it should be stated that TMPRSS2 levels in the transiently transfected 293T cells examined here might exceed those in lung tissue. Moreover, residual entry of the S1/S2 mutant into Caco-2 cells was not facilitated by cathepsin L, as one would have expected from previous work with Calu-3 and Huh7/TMPRSS2 cells^16^, but remained TMPRSS2 dependent. Whether these discrepancies reflect cell line specific differences remains to be determined. In this context, it is noteworthy that Caco-2 cell entry driven by the S protein of SARS-CoV also relied on TMPRSS2 while entry driven by the glycoprotein of Ebola virus, which is known to depend on cathepsin B and cathepsin L activity^30^, was blocked by the cathepsin B/L inhibitor MDL28170 (data not shown). Thus, active cathepsin L seems to be expressed in Caco-2 cells. Finally, our results suggest that the second arginine at the S2′ site might be essential for MERS-S activation by TMPRSS2, since mutation of this residue markedly reduced S protein-driven entry into Caco-2 cells, while mutation of the first arginine did not diminish entry efficiency

Efficient MERS-S-driven entry into Vero E6, 293T and 293T+DPP4 cells was facilitated by cathepsin L and was largely independent of the S1/S2 site but required an intact S2′ site, in keeping with cleavage of this site being required for liberation of the N-terminus of the fusion peptide in the S2 subunit. However, several points are noteworthy regarding this finding. First, mutation of a previously proposed cathepsin L cleavage site^23^ in MERS-S did not diminish viral entry. This site was altered to contain a consensus signal for attachment of an N-linked glycan which was previously shown to abrogate usage of this site^23^. We cannot exclude that the newly introduced glycosylation signal was not used in the 293T cell line employed for particle production. However, production of the mutant in BHK-21 cells resulted in the same entry phenotype (data not shown), suggesting that differential N-glycosylation might not account for the discrepant results of the present study and published work. Second, it seemed that the effects of S2′ site mutation were slightly more profound when entry into 293T as compared to 293T+DPP4 cells was analyzed. This may suggest that high levels of receptor expression might reduce the requirement for proteolytic activation. However, this conclusion is entirely speculative. Third, it is noteworthy that Vero E6, 293T and 293T+DPP4 cell entry driven by the S protein mutant with altered S1/S2 and S2′ sites was only moderately reduced and Vero E6 cell entry remained cathepsin L dependent. This suggests that cathepsin L can use auxiliary S2′ and potentially also S1/S2 sites for S protein activation, a finding recently also documented for SARS-S^31^, and the nature of these sites remains to be determined.

## Materials and Methods

### Plasmids

We employed pCAGGS-based expression plasmids encoding vesicular stomatitis virus (VSV) glycoprotein (VSV-G), wild-type (wt) MERS-S or cleavage site mutants 748 SSVR 751, 748 ASVA 751, 763 NYT 765, 884 ASAA 887 and 748 ASVA 751 + 884 ASAA 887 that have been described previously^10,16,21,23^. Additionally, MERS-S-proteins containing single amino acid exchanges at the cleavage sites of the S1/S2 border, ASVR (R748A) and RSVA (R751A), or the S2′ site, ASAR (R884A) and RSAA (R887A), were generated by overlap extension PCR (S protein mutants are summarized in Fig. 1). For all S proteins, we constructed versions with or without a C-terminal V5 tag. Further, we generated expression plasmids for human TMPRSS2 and human DPP4 N-terminally linked to DsRed (DsRed DPP4) by PCR-based methods and inserted the respective open reading frames and into the pQCXIP vector. In case of the expression vector for TMPRSS2, the vector encoded selection marker for puromycin resistance was exchanged by that for blasticidin resistance taken from the (pcDNA6/TR) vector. The integrity of the PCR-amplified sequences was verified by automated sequence analysis (Microsynth Seqlab).

### Cell culture

293T (human) and Vero E6 (African green monkey) cells were cultivated in Dulbecco’s modified Eagle’s medium (DMEM; PAN Biotech) while the human colorectal adenocarcinoma cell line Caco-2 was grown in Minimum Essential Media (MEM, Life Technologies). All media were supplemented with 10% fetal bovine serum (FBS, PAN Biotech) as well as 1x penicillin and streptomycin from a 100x stock solution (PAN Biotech) and cells were incubated in a humidified atmosphere at 37 °C and 5% CO_2_. Transfection of 293T cells was performed by calcium-phosphate precipitation.

### Protease inhibitors

To test which proteases facilitate MERS-S activation, target cells were preincubated with cathepsin L inhibitor (MDL28170; Sigma-Aldrich) or serine protease inhibitor (Camostat, Sigma-Aldrich) for 2 h, before the cells were inoculated with transduction vectors.

### Antibodies

The following antibodies were used as primary antibodies: Anti V5 (Invitrogen), anti β-actin (Sigma-Aldrich), anti VSV-M (Kerafast). As secondary antibody, an anti-mouse HRP (horse radish peroxidase) conjugated antibody was employed (Dianova). All antibodies were diluted in phosphate buffered saline containing 0.5% Tween20 (PBS T) and 5% skim milk.

### Production of VSV pseudoparticles (VSVpp) and transduction of target cells

We employed a previously described protocol for VSVpp production^25,32^. In brief, 293T cells were transfected with expression plasmids for wild type (wt) or mutant MERS-S, VSV-G (positive control) or empty expression vector (negative control). At 24 h post transfection, cells were inoculated with VSV*ΔG fLuc^24^ (Indiana strain, kindly provided by G. Zimmer) for 1 h at 37 °C and 5% CO_2_. Next, the inoculum was removed, cells were washed with PBS and standard culture media containing an anti VSV-G antibody (produced in I1 hybridoma cells, ATCC CRL-2700) was added to all cells except for those transfected with VSV-G expression vector. The cells were further incubated for 24 h before the VSVpp-containing supernatant was harvested, cleared from cellular debris by centrifugation (3,000 × g, 10 min), and either stored at −80 °C or directly used for transduction experiments. For the latter, target cells were grown in 96-well plates. If necessary, the cells were previously transfected with expression plasmids for DsRed DPP4 and/or TMPRSS2 (24 h in advance), and/or pre-treated with protease inhibitors (2 h in advance). For transduction, the culture medium was aspirated and VSVpp were added to the cells. Transduction efficiency was quantified by measuring the virus encoded firefly luciferase (fLuc) activity in cell lysates using a commercial kit (PJK) and a plate luminometer (Hidex).

### Analysis of MERS-S expression and incorporation into VSVpp

293T cells were transfected with expression plasmids of wt or mutant MERS-S harboring a C terminal V5 tag. For analyses focusing on the S protein activation by TMPRSS2, cells were cotransfected with plasmids for the respective MERS-S construct and TMPRSS2. To investigate S protein expression in target cells, whole cells lysates (WCL) were prepared by lysing the cells in 2x SDS-sample buffer (0.03 M Tris-HCl, 10% glycerol, 2% SDS, 5% beta-mercaptoethanol, 0.2% bromophenolblue, 1 mM EDTA). In the case that incorporation of S proteins into VSVpp was investigated, VSVpp present in cell culture supernatants were pelleted by high speed centrifugation (25,000 × g, 2 h, 4 °C) through a sucrose cushion and lysed in 2x SDS-sample buffer. Following SDS-PAGE under reducing conditions, proteins were transferred onto nitrocellulose membranes (Hartenstein), blocked (30 min, 5% skim milk in PBS-T) and incubated with antibodies targeting the V5 tag (S proteins), ß actin (WCL loading control) or VSV-M (VSVpp loading control), in combination with HRP-conjugated secondary antibodies (Dianova). Western blots were developed using a self-made chemiluminescence reagent in combination with the ChemoCam imaging system and the ChemoStar Professional software (Intas Science Imaging Instruments).

### Quantitative detection of transcripts via quantitative PCR (qPCR)

The relative transcript levels of DPP4, furin, cathepsin L and TMPRSS2 were determined by quantitative PCR (qPCR). For this, total RNA was extracted 293T, Caco-2 and Vero E6 cells by using the RNeasy Mini Kit (QIAGEN GmbH) according to manufacturer’s instructions. Afterwards, 1 µg RNA was DNase-treated (New England Biolabs) and subsequently reverse transcribed into cDNA by using SuperScript™ III First-Strand Synthesis System (Thermo Fisher Scientific) both following the manufacturer’s specifications. In addition, commercially available cDNA (TAKARA) isolated from human lung tissue was analyzed. The qPCR was performed with 1 µl cDNA/reaction employing the QuantiTect SYBR® Green PCR kit (QIAGEN GmbH) on a Rotor Gene® Q (QIAGEN GmbH) platform. All samples were measured in triplicates. Further, serial 10-fold dilutions of expression vectors for ß-actin (ACTB, housekeeping gene control), DPP4, furin, cathepsin L and TMPRSS2 that contained 1 to 10^6^ copies/reaction were measured and used to generate standard curves for ACTB and each target, and to calculate their respective copy number. For normalization, target copy numbers were divided by ACTB copy numbers and are displayed as target gene copies per copy ACTB.
